# UNDERSTANDING THE ROLE OF STRESS PROCESSES IN CUMULATIVE DIS/ADVANTAGE PROCESSES

**DOI:** 10.1093/geroni/igz038.1562

**Published:** 2019-11-08

**Authors:** Deborah Carr

**Affiliations:** Boston University, Boston, Massachusetts, United States

## Abstract

In my book Golden Years (2019), I argue that the psychosocial consequences of normal biological processes of aging are intensified for those who have had lives of disadvantage, just as the harmful consequences of life-course disadvantages are particularly acute for those experiencing age-related physical health declines. In this paper, I discuss the role of stress processes, including stress proliferation and amplification, as possible mechanisms contributing to cumulative dis/advantage. I evaluate these ideas empirically by focusing on the linkages between functional limitations (an indicator of normal biological aging) and psychological well-being in later life, and explore the extent to which these linkages are amplified diverse indicators of life course disadvantage including low education; poor-quality employment; avoidant coping strategies; and family-related strains including intensive caregiving. Analyses are based on data from the Midlife in the United States (MIDUS) study. I discuss the implications of incorporating stress process models in cumulative dis/advantage research.

